# A Rabbit Model for Prolonged Continuous Intravenous Infusion Via a Peripherally Inserted Central Catheter

**DOI:** 10.3389/fphar.2021.637792

**Published:** 2021-04-08

**Authors:** Eyal Dor, Tseela David, Hani Dekel Jaoui, Arieh Schwartz, Tzadok Baruchi, Amram Torgeman, Alon Ben David, Osnat Rosen, Arnon Tal, Amir Rosner, Ran Zichel, Eran Diamant

**Affiliations:** ^1^ Department of Biotechnology, Israel Institute for Biological Research, Ness Ziona, Israel; ^2^ Veterinary Center for Pre-clinical Research, Israel Institute for Biological Research, Ness Ziona, Israel

**Keywords:** PICC, catheter, rabbit, infusion, pump, intravenous, spirometry, model

## Abstract

Medical treatment may require the continuous intravenous (IV) infusion of drugs to sustain the therapeutic blood concentration and to minimize dosing errors. Animal disease models that ultimately mimic the intended use of new potential drugs via a continuous IV infusion in unrestrained, free roaming animals are required. While peripherally inserted central catheters (PICCs) and other central line techniques for prolonged IV infusion of drugs are prevalent in the clinic, continuous IV infusion methods in an animal model are challenging and limited. In most cases, continuous IV infusion methods require surgical knowledge as well as expensive and complicated equipment. In the current work, we established a novel rabbit model for prolonged continuous IV infusion by inserting a PICC line from the marginal ear vein to the superior vena cava and connecting it to an externally carried ambulatory infusion pump. Either saline or a clinically relevant formulation could be steadily and continuously infused at 3–6 ml/h for 11 consecutive days into freely moving rabbits while maintaining normal body temperature, weight, and respiration physiology, as determined by daily spirometry. This new model is simple to execute and can advance the ability to administer and test new drug candidates.

## Introduction

Discovering new drug therapies through the infusion of compounds into the vascular system involves examining the potential effects of drugs in an animal disease model (Nolan and Klein, 2002; Yu, 2011). Part of the predictive power of an animal model depends on how close it resembles the pathophysiology and pharmacology of a human disease and replicates the therapeutic route and dosing. To ultimately mimic human pharmacokinetics (PK), as well as pharmacodynamics (PD), continuous infusion of therapeutic compounds, such as antimicrobials and small molecule inhibitors that suffer from rapid clearance, should be utilized in animal models (Zhao et al., 2016).

Long-term continuous IV infusion systems comprise a flexible tubular intravascular catheter connected to an infusion pump. Although various infusion systems for long-term vascular access have been used for several decades in preclinical studies with different animal models, from small rodents to horses (Lemmel and Good, 1971; Burrows, 1973; Burt et al., 1980; DaRif and Rush, 1983; Aguiar et al., 1988; Sellon et al., 2001; Laliberte et al., 2005; Turner et al., 2011), both catheterization techniques and delivery systems still pose major challenges.

To allow animals to move freely during the continuous infusion period, several methods are considered. For limited infusion volumes, the pump can be implanted subcutaneously and then directly connected to the catheter. For larger infusion volumes, the pump can be placed outside the cage and tethered to the animal using a specialized swivel. Another ambulatory technique involves the use of a carried pump, considering the appropriate ratio between the weight of the pumping system and the infused animal (Francis et al., 1992; Towell et al., 1992; Kung et al., 1994; van Wijk et al., 2000; Nolan and Klein, 2002; Mage et al., 2017).

Various central venous access methods for long-term continuous infusion are being used in preclinical studies as well as in the clinic. To achieve the prolonged, sustained infusion flow of drugs and other compounds and to decrease the need for recurrent needle indwelling, the most common method is the cannulation of a large central vein, usually close to the heart. Accessing a central vein is enabled either by a proximal central vein or a peripheral vein, which are categorized accordingly as two main methodologies: 1) Central venous catheterization (CVC), also known as a central line (CL), in which the target vein, most commonly the superior or anterior vena cava, is accessed via a proximal central vein, such as the internal jugular, subclavian or femoral vein (Smith and Nolan, 2013); 2) A peripherally inserted central catheter (PICC), a minimally invasive device by which a venous catheter is inserted percutaneously into a peripheral vein and terminates in central veins (Racadio et al., 2001; Chopra et al., 2012). CL and PICC enable access to a large vein for the delivery of medication, as well as for laboratory testing and monitoring (Chopra et al., 2012). Nevertheless, while both CL and PICC may have some serious medical complications, such as microbial colonization, CL may also involve infections associated with securing sutures (Karpanen et al., 2019). In addition, among traditional central venous catheters, which should be typically removed in two weeks, PICCs are exceptional in that they can provide durable venous access for weeks (and even months) to administer medications and blood products, as well as to enable blood sampling. PICCs also avoid many of the technical complications associated with traditional central venous catheter placement (Gibson et al., 2013; Chopra et al., 2014). Owing to their relatively safe long-term intravascular access, comfort, and ease of transition to home therapy, PICCs are popular in children, infants and preterm infants (Racadio et al., 2001; Ainsworth and McGuire, 2016). PICCs can be made of either silicon rubber to lower the thrombosis risk or polyurethane, a tougher material that enables thinner lumen walls with larger internal diameters of the lumina, thereby increasing flow rates and reducing the catheter rupture potential (Bishop et al., 2007). In comparison to alternative peripheral access, in which the catheter tip resides in a peripheral vein, PICCs eliminate the discomfort associated with recurrent phlebotomy and vein changes, and provide extended and reliable venous access (Chopra et al., 2012).

Surprisingly, even though PICCs have been in use in the clinic for decades, we could not find even one study regarding an animal disease model with long-term continuous infusion based on PICC. Rather, all animal models that use a continuous infusion apply other CL methodologies that require surgical procedures with general anesthesia.

In the current study, a novel rabbit model of long-term continuous IV infusion based on PICC and spirometry monitoring was developed. The catheter was inserted via the marginal ear vein and terminated in the superior vena cava. Following PICC installation, either saline or a clinically relevant formulation, used to dissolve therapeutic small molecules with suboptimal water solubility, was continuously infused for 11 days using ambulatory pumps harnessed to the back of each rabbit. Respiration was individually monitored prior to and during the infusion period using a spirometer. This mode of respiration monitoring in unrestrained rabbits has recently been demonstrated to be an efficient quantitative tool to detect the manifestation of botulism symptoms (Diamant et al., 2018; Torgeman et al., 2018).

To the best of our knowledge, the rabbit model of long-term PICC-based continuous IV infusion with respiration monitoring detailed herein is the first report of accessing the vena cava through the marginal ear vein in a free roaming animal model while maintaining a continuous infusion. This new rabbit infusion model, combined with spirometry, can serve as a reliable setup to test new drugs, especially with respect to respiration monitoring.

## Materials

### Catheter

Premicath, 28 G (1 Fr), a 20 cm long, peripherally inserted central venous polyurethane catheter with a 24 G splitting needle, either with a stylet (cat. 1,261.203, VYGON, France) or without a stylet (cat. 1,261.203, VYGON, France).

### Infusion Pump

SAI 3D minipumps (SAI Infusion Technologies, United States) with sterile 50 ml syringes (cat. CRN/50, SAI Infusion Technologies, United States).

### Infusion Materials

Either saline or a clinically relevant formulation based on NaCl, Solutol, and benzylalcohol (Sol/Benz).

### Spirometry System

The inhalation parameters were recorded by a computer-monitored spirometry system. The system consisted of a snout-only mask connected to a T-type nonrebreathing two-way valve. A model 4,100 Thermal Mass Flowmeter (TSI Inc. Shoreview, MN, United States) with low flow resistance was connected to the valve’s inhalation port.

## Methods

### Rabbits

Ten healthy New Zealand White rabbits provided by Charles River (Lyon, France) were used in this study. All rabbits were females weighing between 2.5 and 4.5 kg.

#### Ethics Statement

Experiments were approved by the IIBR Animal Care and Use Committee and were maintained in accordance with the guidelines of the care and use of laboratory animals published by the Israeli Ministry of Health (protocols #RB-12–18 and #RB-21–19). All efforts were made to minimize animal suffering. All animals were observed for morbidity and mortality, overt signs of toxicity (including abstinence of food and water), and any signs of distress throughout the study. Rabbits were given free access to food and water throughout all experiments.

### Harnessing the Pumps

Each pump was harnessed to the rabbit’s back by placing it in a flexible pocket adjusted from an armband (originally intended to carry smartphones) to ease the sliding of the pumps in and out of the pocket. The pocket was fastened to a specialized rabbit jacket (Lomir Cat. BJ02) using cable ties.

### Inhalational Data

Respiratory data were collected and analyzed as detailed previously (Diamant et al., 2018; Torgeman et al., 2018). Briefly, Rabbits were acclimated to spirometry measurements for several days while carrying minipumps on their backs with disconnected syringes filled with 50 ml of water (imitating the conditions during the infusion period). During measurements, un anesthetized rabbits respired freely. The data collected from naive animals were in line with published values of normal rabbit respiration physiology (Bide et al., 2000). Minute volume (Mv) data were collected at 20 millisecond (ms) intervals (500 data points in 10 s) and were analyzed using Microsoft Excel (2013). Accordingly, 13 independent measurements collected over 13 consecutive days while carrying the pumps prior to the continuous infusion were used to calculate the individual mean and standard deviation (SD) of the Mv values. The confidence limits were determined as the mean ± 2*SD (Diamant et al., 2018).

### Peripherally Inserted Central Catheter Insertion and Continuous Infusion

During the procedure, rabbits were anesthetized with 3% isoflurane with a facemask (UNO, Netherlands). Insertion of the PICC was performed under aseptic conditions to prevent phlebitis. The skin over the vein was swabbed with 70% alcohol. The splitting needle was inserted into the marginal vein of the right ear, and then, the catheter was slowly inserted through the needle using sterile forceps until reaching the superior vena cava, proximal to the heart. The needle was then removed, and the catheter was immediately flushed with 3 ml of heparinized saline (1 unit/ml) and secured to the auricle skin with Leucoplast adhesive straps. The catheter was then instantly connected to a 50 ml syringe of the minipump using two joined extension lines of 30 cm each (CN-2030, Biometrix, Netherlands), and a saline infusion at a rate of 3 ml/h was started. Verification of the exact location of the catheter tip was assessed by radiography. Soft X-ray images were taken using an Orange 1040 HF portable X-ray unit (EcoRay, Seoul, Korea) tuned to 64 KVP with 4 mas. Images were developed and analyzed using a Point-of-Care CR 360 system (CareStream Health, Inc. NY, United States). Following radiography, the 50 ml syringes containing either saline or Sol/Benz were replaced twice a day: The infusion rate was set to 6 ml/h for 8 h (during the daytime) and then to 3 ml/h for 16 h (at night). During the continuous infusion phase, just before the end of the complete infusate consumption, the empty syringes were replaced with new full 50 ml syringes, an operation that caused the infusion to pause for no longer than 5 min, to enable the plunger to move backwards. In the meantime, a bolus of 2 × 1 ml of heparinized saline (1 unit/ml) was slowly injected via the extensor set to prevent clogging.

### Determining Indocyanine Green Serum Levels

At day 11, just prior to cessation of the infusion, 1.5 ml of 1.0 mg/ml ICG (IC-GREEN™, indocyanine green for injection, USP, cat. NDC 17478–701–02, Akorn Inc. IL, United States) was injected via the catheter by disconnecting the syringe and using the extensor directly. Immediately after the ICG injection, 2 ml of saline was injected to flush the extensor. One minute and 3 min following the ICG injection, blood was collected from the unoccupied, opposite marginal ear vein, and serum was separated using BD Vacutainer serum separation tubes (cat. 367812, BD, United States). Serum fluorescence was measured using an Infinite M200 instrument (Tecan, Switzerland) at excitation wavelength of 805 nm and an emission wavelength of 835 nm. A standard curve was prepared by 2-fold dilutions of ICG in naïve rabbit serum, from 24 mg/L to 0.09375 mg/L. Data extrapolation and back calculation of the ICG serum concentration were performed using GraphPad Prism (GraphPad, United States).

### Sol/Benz Formulation Preparation

A solution of 0.675% NaCl in water for injection (WFI) was prepared in advance. One percent Solutol (BASF Kolliphor HS 15, 2019, Sigma, 42966) was prepared by adding the 0.675% NaCl solution to premolten Solutol, stirring for 5 min, sonicating for 5 min, and stirring again for 5 min. Benzyl alcohol (cat. 1.00987.2500, Merck KGaA, Germany) was transferred into a sterile vessel; then, 1% Solutol in the 0.675% NaCl solution was slowly added, and vigorous stirring was carried out for 20 min. The formulation was then filtered through 0.2 µm filters and transferred in a sterile hood to the SAI 50 ml pump syringes.

### Statistics

To compare the Mv value means of the rabbit groups that were infused either with saline or Sol/Benz and the Mv value means of the rabbit groups before and after infusion, *t*-tests were performed, and *p* values were calculated. The distribution of the individual Mv values, computed by the D'Agostino-Pearson’s omnibus K2 normality test, did not deviate from the Gaussian distribution in terms of asymmetry and shape. All of the statistical analyses were done using GraphPad Prism (GraphPad, United States).

## Results

The main objective of the current study was to establish a rabbit model for prolonged continuous IV infusion using a PICC-based technique combined with daily spirometry. The new model allows for the continuous administration of novel drug candidates, together with respiration monitoring, and is relevant to a wide variety of diseases, especially, but not restricted to, those that involve pulmonary distress.

### Peripherally Inserted Central Catheter Insertion and Continuous Infusion

**FIGURE 1:**
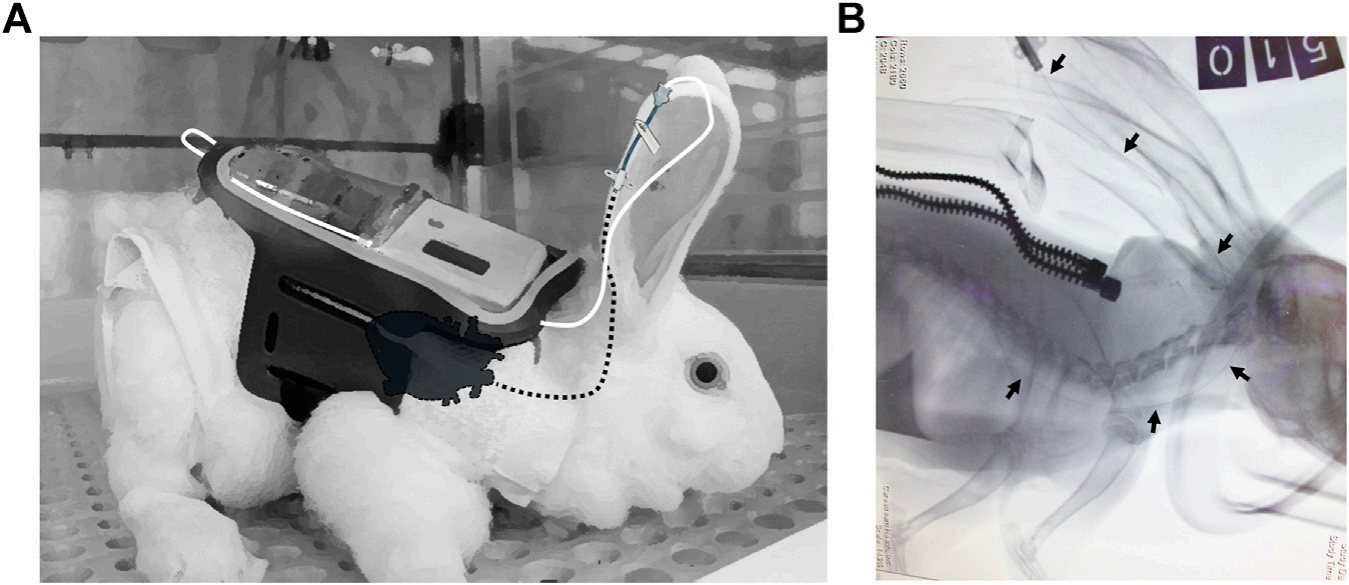
PICC insertion **(A)** Illustration of the connection between the pump and catheter. The carried pump is harnessed to the back of the rabbit by an external designated jacket (white) and a flexible pocket adjusted from an armband (black). The external tip of the PICC is connected to the syringe by a connecting line, consisting of two joined flexible plastic extensors (white line), and the PICC is inserted from the marginal ear vein to the superior vena cava, proximal to the heart (dashed black line, designating internal parts). The adhesive tape that prevents the removal of the PICC is not illustrated **(B)** X-ray imaging of the PICC from the marginal ear vein to the superior vena cava. Rabbits were catheterized under isoflurane anesthesia (the gas mask was imaged). Soft X-ray images were taken at a left lateral posture using an Orange 1040 HF portable X-ray unit tuned to 64 KVP with 4 mas. Images were developed and analyzed using a Point-of-Care CR 360 system. The black arrows show the catheter guide wire location from the marginal ear vein to the superior vena cava (the heart is shadowed). The catheter guide wire was left for the sake of imaging; it was removed afterward before initiating the infusion. The image was taken while the rabbit was carrying the jacket (the zipper is shown).

Seven rabbits were aseptically catheterized through the right marginal ear vein at an average time of 20 min per rabbit. The procedure was performed under isoflurane anesthesia. X-ray imaging enabled us to assure that the tip of the catheter was located within the superior vena cava (Figure 1). Immediately following PICC insertion, the catheter was aseptically connected to a carried minipump, and a continuous infusion of either saline or Sol/Benz was initiated at 3–6 ml/h and maintained for 11 consecutive days (four rabbits were administered saline, and three rabbits were administered Sol/Benz). The 50 mL syringes containing either saline or Sol/Benz were replaced twice a day: every morning, the infusion rate was set to 6 ml/h for 8 h (8:00–16:00), and every evening, it was set to 3 ml/h for 16 h (16:00–8:00). Flow rates were changed to test both relatively high and low rates, and hence to define a working range. The transition from a high flow rate to a low flow rate and backwards served to simulate infusion regimens that require rate adjustments during infusion to optimize drug efficacy.

During the 11-days infusion period, two clogging events were recorded: one on the fifth day (a saline-infused rabbit) and another on the seventh (a Sol/Benz-infused rabbit). Both events could technically be attributed to chewing damage leading to leakage through the plastic extensor between the pump and the ear.

**FIGURE 2:**
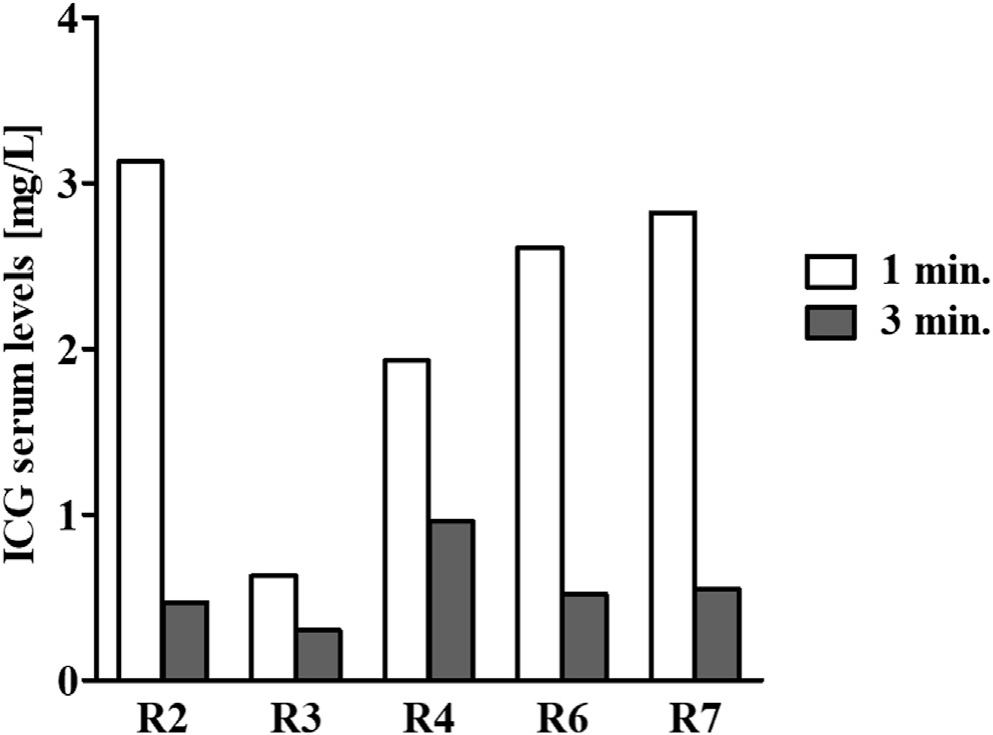
ICG serum level determination. At the end of the continuous 11-days infusion period, 1.5 ml of 1.0 mg/ml ICG was injected via the catheter. Next, blood samples were collected from the unoccupied marginal ear vein one (white bars) and three (gray bars) minutes following an ICG injection. Serum samples were prepared, and fluorescence was measured to determine the ICG serum levels. For all tested rabbits, the ICG serum levels at one and 3 min matched the anticipated concentrations according to its known half-life of approximately 150–180 s. Due to technical difficulty, the first bleed of rabbit #506 was carried out not according to the planned schedule but at ∼2 min following the ICG injection. Hence, the serum levels were lower at that point in time.

To ensure that the catheters were properly located in the veins, at day 11 of the infusion, just prior to termination of the infusion, indocyanine green (ICG) was injected via the catheter of the infused rabbits, and serum fluorescence was measured to determine the ICG serum levels (Figure 2). For all tested rabbits, the ICG serum levels at one and 3 min matched the anticipated concentrations according to the known half-life of ICG of approximately 150–180 s (Cherrick et al., 1960).

### Spirometry and Other Vital Physiological Parameters

**FIGURE 3:**
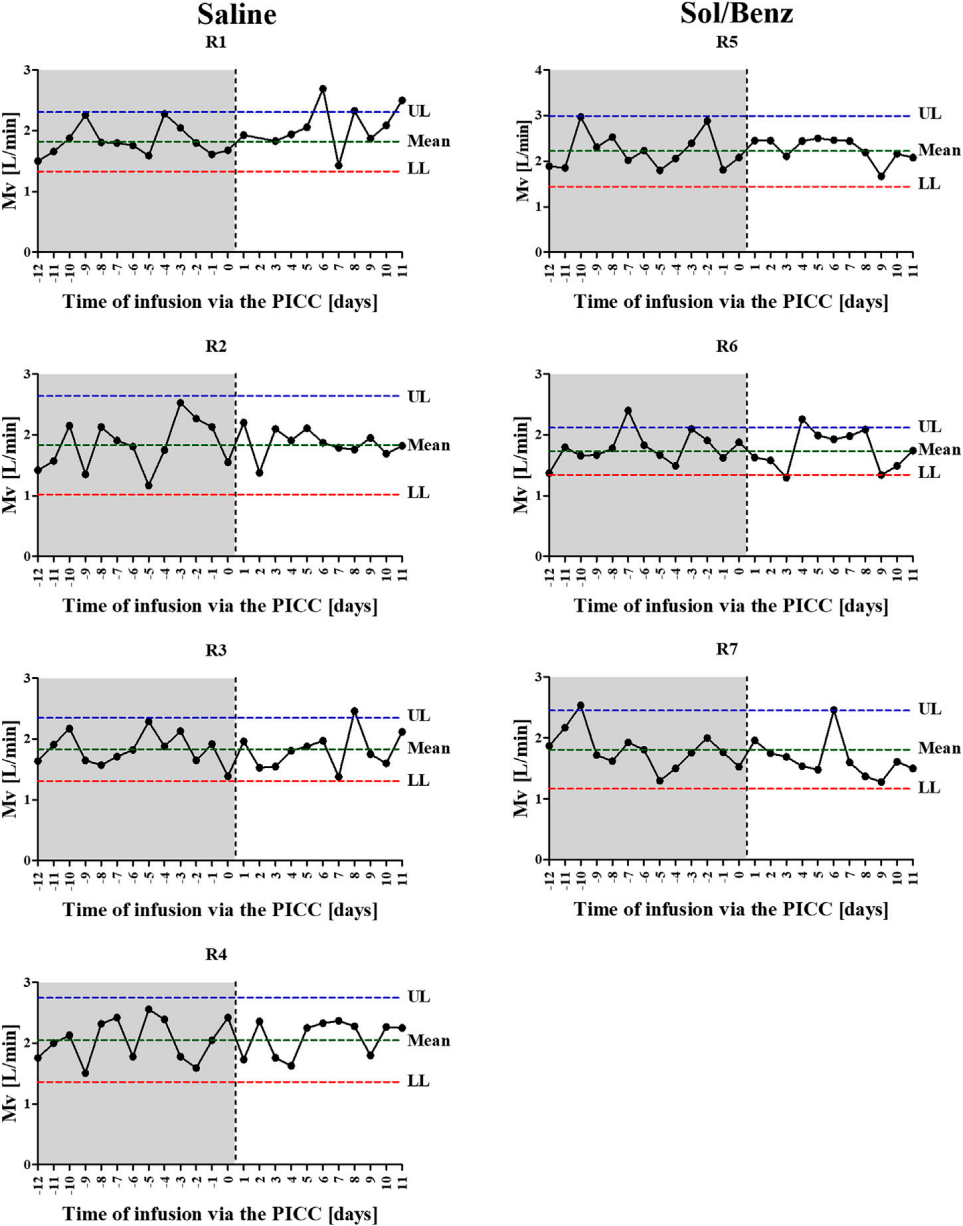
Individual Mv values before and during the IV continuous infusion. Following acclimation to carrying the minipumps, 13 independent measurements were collected over 13 consecutive days and were used to calculate the individual normal mean and standard deviation of Mv values (preinfusion period: area in gray). Following PICC insertion (the time point is designated by the vertical dashed line) and during the 11-days IV continuous infusion, the Mv values were determined once per day (infusion period: area in white). Left panel: saline infused rabbits. Right panel: Sol/Benz infused rabbits. The confidence limits were determined as the mean ± 2 * SD.

**FIGURE 4:**
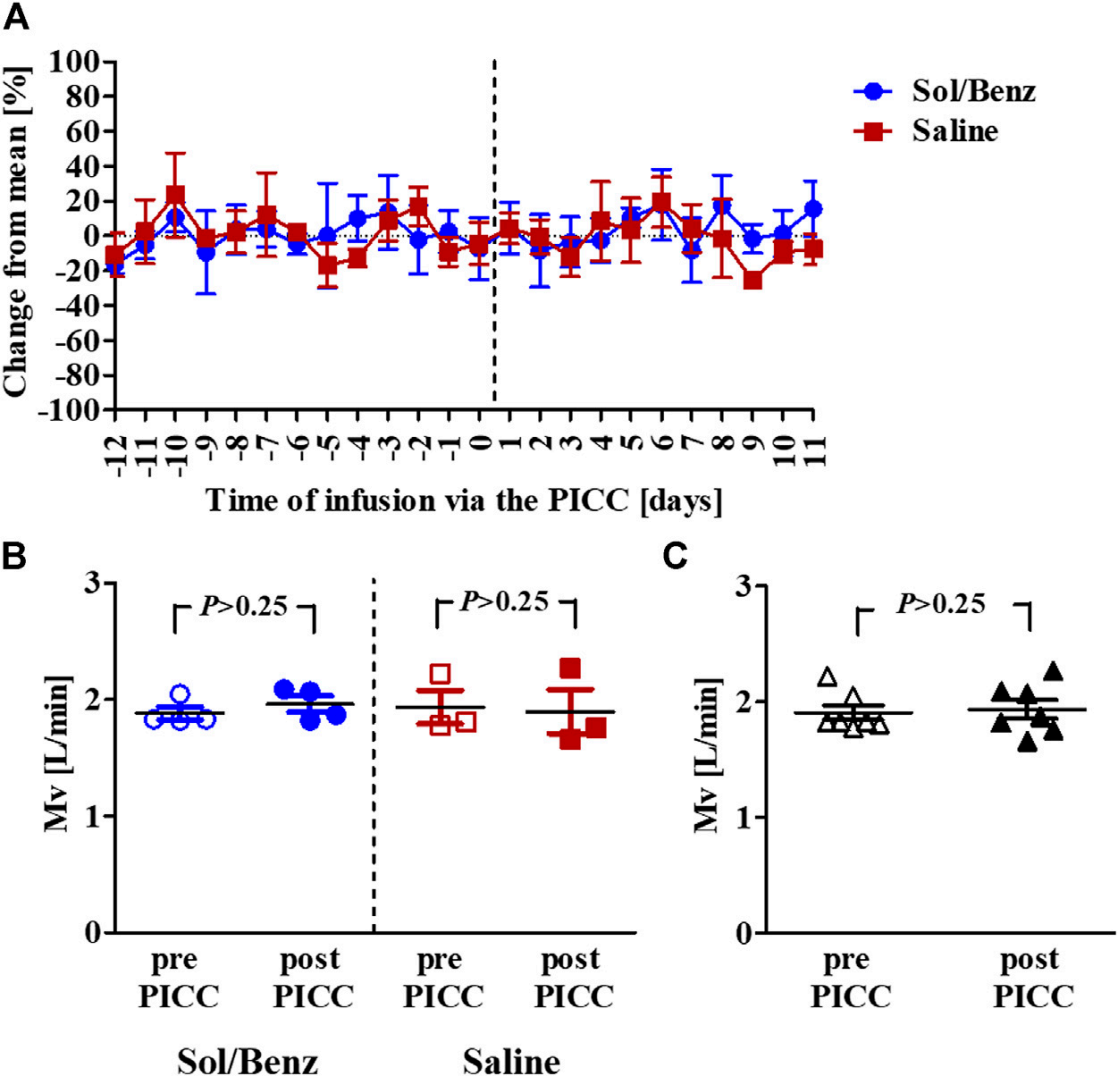
Mean Mv values before and during the IV continuous infusion **(A)** Mean Mv values of rabbits infused with either saline (red squares) or solvent (blue circles). The vertical dashed line designates the PICC insertion time point **(B)** Mean Mv values before (empty signs) and after (whole signs) PICC insertion within each group. The blue circles designate solvent infusion, and the red squares designate saline infusion **(C)** Mean Mv values pre- (empty rectangles) and post- (whole rectangles) PICC insertion for all seven rabbits. *p* = *p value*.

Spirometry measurements of acclimated rabbits were collected over 13 consecutive days and were used to determine the confidence limits (the mean ± 2*SD of the preinfusion Mv values) (Diamant et al., 2018). Following PICC insertion, the respiration parameters were individually monitored for 11 days. The mean Mv did not change during the infusion period compared to the preinfusion period in either of the two test groups (saline and Sol/Benz) or between these two groups (Figures 3, 4).

**FIGURE 5:**
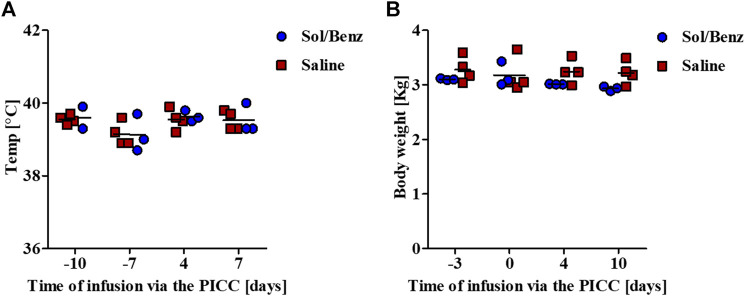
Monitoring vital physiological parameters. Body temperature and weight were measured prior to and during the continuous infusion period **(A)** Body temperature was measured rectally **(B)** Rabbits were measured while carrying all the infusion equipment. The net weights were calculated by subtracting the weight of the pumps, including the reservoirs, jackets, and pockets.

Body temperature and weight were monitored before PICC insertion and during the 11-days infusion period. No change in average body temperature or body weight was observed during the experiment (Figure 5).

Following termination of the infusion, all rabbits were sacrificed. The liver, heart, and lungs were examined by postmortem autopsy. No changes in size, shape or color were observed. Examination of the vascular morphology of the catheterized veins showed normal tissue, without any signs of vessel damage, bleeding or necrosis.

## Discussion

Medical research is facilitated through translational science, i.e., *in vitro* techniques followed by a preclinical investigation involving animal models (Yu, 2011). In the pursuit of novel effective drug therapies, it is vital to design animal disease models that will ultimately mimic the intended use of the new potential drug and all its aspects. In case that PK studies show that the drug must be continuously infused to enable a flexible therapeutic regimen and sustain an efficient drug concentration in circulation, an adequate animal model of continuous infusion needs to be developed and used.

The model described in the current study was developed in rabbits for the following reasons: rabbits are used as animal models for human diseases in various medical research fields, including metabolic disorders, cardiovascular diseases, inflammatory responses, chronic pancreatitis, infectious diseases, and more (Shiomi, 2009). Further, rabbits are the third most commonly used species for experimental research in the European Union (Hedenqvist et al., 2013). Rabbits are larger than mice and rats and are the animal of choice in many studies, and it is easier to establish an animal model that involves continuous infusion in rabbits than in mice and rats (Weber et al., 2011). Rabbits are phylogenetically closer to humans than rodents. Due to their anatomical, physiological, genetic, and biochemical similarities to humans, rabbits are preferentially used in pulmonary, cardiovascular, and metabolic studies (Kamaruzaman et al., 2013).

In the current work, a new rabbit model for prolonged continuous IV infusion using a PICC line and an ambulatorily carried battery-powered pump combined with daily spirometry was established. We were able to continuously infuse rabbits for at least 11 consecutive days with two different clinically relevant formulations to simulate the infusion of both water-soluble (saline) and water-insoluble (Sol/Benz) compounds at 3–6 ml/h. The surfactant Solutol is a water soluble nonionic solubilizer and emulsifying agent intended for use in parenteral formulations with lipophilic compounds to solubilize and stabilize them at a desired concentration (BASF Kolliphor HS 15, 2019; Strickley, 2004). An aqueous formulation of Solutol and the organic solvent benzyl alcohol can be used for the enhancement of solubilization of poorly water-soluble drugs (Kim et al., 2008; Lee et al., 2016). During the 11-days infusion period, two clogging events were recorded, and both could technically be attributed to leakage through the plastic extensor between the pump and the catheter due to chewing. Following puncture of the plastic extensor, the infusate could not flow into the vein; thus, blood could flow backward and clog the catheter. These events could be prevented by better securing the pumps in their jackets and protecting the extensors. Such events could be corrected by instant catheterization of the marginal vein of the other ear, as was later successfully applied in another experiment of continuous infusion using the PICC-based rabbit model. Just before the end of the infusion period, ICG was injected via the catheter of each of the infused rabbits, and the fluorescence intensity of serum collected from the unoccupied marginal ear vein at one- and 3-min intervals was measured. The results correlated well with the anticipated t_½_ of ICG (18% per minute (Cherrick et al., 1960)), indicating the patency of the catheter and proving that the infusate flowed systemically through the vein. All the measured physiological parameters were normal throughout the experiment, including body temperature, weight, and respiration, regardless of the type of infused formulation (Sol/Benz or saline).

Our new model involves neither surgery nor complicated tethering parts and can hence be easily performed in unrestrained, unanesthetized rabbits. Furthermore, the fact that this is a noninvasive procedure implements the refinement principle, one of the Three Rs guiding principles for the ethical use of animals in research, which practically means less pain and suffering for animals during the procedure (Tannenbaum and Bennett, 2015). Other publications regarding a continuous infusion in a rabbit model can be found in the literature. In all of these published studies, until now, either an ambulatory pump was tethered to a peripheral venous catheter (Beckel, 1962; Hattori, 1964; Bugnon et al., 1998; Mustola et al., 2000; Robaux et al., 2001; Jacqueline et al., 2002; Li et al., 2012; Mage et al., 2017) or a central line catheter (Towell et al., 1992; Barrow and Guyot, 1996) with a swivel or an implanted pump was connected to a central line catheter (Esper et al., 1987; Walsh et al., 1992) or to the jugular vein (Ohar et al., 1990). However, both the implanted pump methodology and central line catheter techniques necessitate surgical procedures that require the work of highly skilled veterinarians and laboratory teams, as well as specialized surgical rooms, and may lead to dangerous complications in animals, such as infection and thrombosis. In contrast, our new rabbit model combines simple techniques: minipumps are carried on the backs of rabbits in light harnessing jackets for long periods of time, enabling them to move and eat freely in their cages without tethers or swivels, and the infusion rate can be easily tuned by using either on-pump buttons or even a Bluetooth remote control. In addition, the PICC line is placed in the superior vena cava via the marginal ear vein within minutes without any surgical intervention, and rabbits recover almost instantly after termination of isoflurane anesthesia. Moreover, anesthesia may not be needed at all if the rabbits are well acclimated and calm.

To the best of our knowledge, the current work is the first to use a PICC line in rabbits for the purpose of a prolonged continuous infusion. Other studies involving a PICC line (Allan et al., 2012; Zhang et al., 2014; Jamal et al., 2015; Aguilera-Correa et al., 2020; Huang et al., 2020), as well as CL (Rodrigues et al., 2015) in rabbits have thus far only aimed to investigate the adverse outcomes of using these catheters, such as inflammation, infection, fibrillation, and thrombosis, by monitoring lesions and other histological alterations. While some of these studies have used the marginal ear vein (Aguilera-Correa et al., 2020; Huang et al., 2020) others either indwelled the Basilic vein in the upper limb (Zhang et al., 2014) or the jugular vein (Jamal et al., 2015; Rodrigues et al., 2015) or inserted the PICC line subcutaneously (Allan et al., 2012).

To establish the new PICC-based technique, our study included several preliminary experiments. First, one rabbit was catheterized with a PICC set that did not include a stylet. As a result, the insertion of the extremely flexible catheter was challenging, and although intended to be imaged using X-ray, we did not observe the exact location of the catheter tip. Consequently, in a follow-up experiment, one rabbit was catheterized with a PICC set that included a stylet (the same model that was used for seven more rabbits in the currently described expanded study). The PICC was connected to a 50 ml syringe of a carried battery-powered minipump, and continuous infusion of saline was conducted for 14 consecutive days (6 ml/h between 8 am and 4 pm; 3 ml/h between 4 pm and 8 am). X-ray imaging was successfully used to confirm the position of the catheter within the superior vena cava prior to infusion while the stylet wire, made of radio-opaque material, was still inside the catheter (data not shown). A postmortem autopsy confirmed that the catheter was correctly placed in the superior vena cava 14 days after its insertion. In addition, no signs of inflammation were observed in the vein. Furthermore, as part of our efforts to establish the new PICC-based method, we wanted to compare the marginal ear vein to the basilic vein by following the PICC method described by Zhang et al. (2014) by using one rabbit. However, exposure of the extremely thin basilic vein, located deep in the upper limb and thus requiring removal of the fur by tedious shaving, seemed much more complicated and cumbersome than the marginal ear vein. Further, the vascular system connecting the basilic vein to the vena cava is heavily branched, so insertion of the catheter may become much more challenging compared to using the marginal ear vein.

The simplicity of using a PICC-based prolonged continuous infusion via the marginal ear vein, together with the power of daily spirometry in unrestrained rabbits, can serve as a valuable tool in various research fields, for example, when respiration monitoring is needed. Recently, we established a rabbit spirometry model to monitor respiration and control the treatment timing following exposure to botulinum toxins (BoNTs), the most poisonous substances known in nature (Diamant et al., 2018; Torgeman et al., 2018). Spirometry was shown in these studies to serve as an accurate, quantitative, and objective means to detect early symptoms of botulism in rabbits, and deviation from the normal minute volume (Mv) following exposure to BoNT was found to be the earliest symptom to be measured among other spirometry-related parameters. Accurate quantification of the postintoxication Mv decline by a spirometer qualified the Mv symptom to become a reliable clinical symptom of botulism (Diamant et al., 2018; Torgeman et al., 2018). Currently, we are involved in an effort to test a small molecule inhibitor (SMI) against BoNT by continuous infusion using our PICC-based rabbit model via the marginal ear vein. To date, 12 rabbits have been infused with a new compound dissolved in a clinically relevant formulation (Sol/Benz) for 10 days, with only a few occlusion events that were instantly resolved by catheterizing a marginal vein of the other ear. In comparison to using peripheral venous catheters, the PICC-based technique allowed us to sharply reduce the frequency of occlusion events that demanded the use of another vein.

The combination of a convenient peripheral access with prolonged continuous infusion by carried pumps may be applied to additional laboratory animals, such as pigs and minipigs, which are used as animal models for human diseases in many translational studies. Particularly, the pig is the most prevalent large animal species for scientific purposes in the European Union (Report from the Commission to the European Parliament and the Council, 2019). Previous studies in pig models that aimed to simplify percutaneous insertion of central venous catheters, and to minimize invasiveness, required cumbersome techniques, such as magnetic and ultrasound guidance (Gonzales et al., 1999; Pinkernelle et al., 2009; Larsson et al., 2015; Furbeyre and Labussiere, 2020). However, these studies established protocols only for sequential blood sampling and for bolus dosing, and in some occasions, incisions were required, too. Notably, none of these studies applied the IV catheters for continuous infusion. Hence, based on the concepts of the current rabbit study, developing a pig model for prolonged continuous IV infusion via a PICC may be feasible.

In conclusion, our new rabbit model of marginal ear vein PICC-based prolonged continuous infusion can be used in different research fields to test new potential therapeutic compounds. The ability to infuse a clinically relevant formulation that can potentially be used as a vehicle for small molecules with low solubility for long periods increases the odds of success.

In addition to botulism, potential drug therapies for other diseases, with an emphasis on the involvement of respiration malfunctions, can be tested using our new rabbit model of continuous infusion. These include new therapies for asthma and other lung diseases, lung injury, and bacterial and viral infections that might cause, among other pathologies, acute respiratory distress syndrome (ARDS) (Keir and Page, 2008; Matute-Bello et al., 2008; Kamaruzaman et al., 2013; Peng et al., 2015; Dehnad et al., 2016; Strandberg et al., 2016). As severe cases of COVID-19 are characterized by life-threatening lung injury and ARDS (Marini and Gattinoni, 2020), our new rabbit model may also be used to test potential new drugs that may reduce lung injury as part of the treatment of COVID-19 patients.

## Data Availability

The original contributions presented in the study are included in the article/Supplementary Material, further inquiries can be directed to the corresponding author.
